# Social environment influences microbiota and potentially pathogenic bacterial communities on the skin of developing birds

**DOI:** 10.1186/s42523-024-00327-2

**Published:** 2024-08-15

**Authors:** Ester Martínez-Renau, Antonio M. Martín-Platero, Kasun H. Bodawatta, Manuel Martín-Vivaldi, Manuel Martínez-Bueno, Michael Poulsen, Juan José Soler

**Affiliations:** 1 Departamento de Ecología Funcional y Evolutiva, Estación Experimental de Zonas Áridas (CSIC), 04120 Almería, Spain; 2 Departamento de Microbiología, Universidad de Granada, 18071 Granada, Spain; 3 Unidad Asociada (CSIC): Coevolución: Cucos, Hospedadores y Bacterias Simbiontes, Universidad de Granada, 18071 Granada, Spain; 4 Natural History Museum of Denmark, University of Copenhagen, Copenhagen, Denmark; 5 Section for Molecular Ecology and Evolution, Globe Institute, University of Copenhagen, Copenhagen, Denmark; 6 Departamento de Zoología, Universidad de Granada, 18071 Granada, Spain; 7 Section for Ecology and Evolution, Department of Biology, University of Copenhagen, Copenhagen, Denmark

**Keywords:** Avian skin microbiome, Bacterial community, Brood parasitism, Social transmission, Eurasian magpie, Great spotted cuckoo

## Abstract

**Background:**

Animal bacterial symbionts are established early in life, either through vertical transmission and/or by horizontal transmission from both the physical and the social environment, such as direct contact with con- or heterospecifics. The social environment particularly can influence the acquisition of both mutualistic and pathogenic bacteria, with consequences for the stability of symbiotic communities. However, segregating the effects of the shared physical environment from those of the social interactions is challenging, limiting our current knowledge on the role of the social environment in structuring bacterial communities in wild animals. Here, we take advantage of the avian brood-parasite system of Eurasian magpies (*Pica pica*) and great spotted cuckoos (*Clamator glandarius*) to explore how the interspecific social environment (magpie nestlings developing with or without heterospecifics) affects bacterial communities on uropygial gland skin.

**Results:**

We demonstrated interspecific differences in bacterial community compositions in members of the two species when growing up in monospecific nests. However, the bacterial community of magpies in heterospecific nests was richer, more diverse, and more similar to their cuckoo nest-mates than when growing up in monospecific nests. These patterns were alike for the subset of microbes that could be considered core, but when looking at the subset of potentially pathogenic bacterial genera, cuckoo presence reduced the relative abundance of potentially pathogenic bacterial genera on magpies.

**Conclusions:**

Our findings highlight the role of social interactions in shaping the assembly of the avian skin bacterial communities during the nestling period, as exemplified in a brood parasite—host system.

**Supplementary Information:**

The online version contains supplementary material available at 10.1186/s42523-024-00327-2.

## Background

Animal hosts maintain diverse and complex microbial communities in both internal and external body regions. These symbiotic microbiotas play important roles related to host evolution and ecology [1–4] through facilitating a myriad of essential functions related to development [5–7], nutrition [8, 9], immunity [10–13], and even chemical communication [13–15]. The microbiota associated with animals also includes potential pathogens that may infect hosts and/or shape community compositions, both of which carry potential negative consequences for host health and fitness [16–18]. Assemblies of these microbial communities can be influenced by a plethora of evolutionary and ecological factors, such as host phylogeny [19–21], diet [22–24], environment and geography [25–27], and social interactions (i.e., direct physical contact with conspecifics) [28–33]. However, the impact of these factors differs by animal host and depends on whether the microbial communities are internal (i.e., gut) or external (i.e., skin) [34]. Assemblies of external microbiota, such as on the skin, feathers, or hair, are particularly vulnerable to colonization by microorganisms from the environment or from con- or heterospecifics that focal individuals interact with (social transmission).

The social environment (i.e., environmental characteristics of interacting individuals) has been suggested to promote similarities in bacterial communities between interacting animals [28–31]. Thus, it should be important in driving similarities in microbiome-derived physiological and behavioural traits of hosts, as well as explaining susceptibility to parasitism [1, 35]. Most evidence for effects of the social environment on the microbiome comes from experimental approaches in a few captive animal models, or from correlational studies in gut and skin microbiota of humans [31, 36–38], non-human primates [28, 29, 32, 39–41], other mammals [42, 43], birds [44–47], amphibians [48], and arthropods [49, 50]. Despite evidence supporting associations between social interactions and the host microbiota, we are just starting to understand how social interactions structure host microbial communities. This is partly due to the confounding effects of sharing environments in the absence of social interactions and the role of host genetics [32]. Within social groups, individuals are likely to share early life environmental conditions, physiological stress, similar resources, diet, and/or genetic relatedness (family groups). Similarities in microbial communities among individuals would be predicted in such cases even in the absence of social interactions. Thus, to understand the role of social interactions, it is essential to disentangle these effects, and studying unrelated individuals interacting under identical environmental conditions may help achieve this [51–54].

In the present study, we take advantage of the brood parasite – host system formed by great spotted cuckoos (*Clamator glandarius*) (hereafter cuckoos) and Eurasian magpies (*Pica pica*) (hereafter magpies). Cuckoos are obligated brood parasites that in Europe mainly lay their eggs in magpie nests, where magpie adults incubate the eggs and take care of the cuckoo chicks during the nestling and fledgling periods [55]. Adult cuckoos thus do not have contacts with their own nestlings, restricting microbial transfers from parents to offspring to the pre-laying phase. When the eggs hatch and parasitic nestlings do not outcompete host nestlings, host adults rear their own nestlings along with parasite nestlings [55]. In these cases, the skin and feathers of parasitic and host nestlings are in close contact, which allows exploring similarities in bacterial communities of natural and foster siblings that cannot be explained by relatedness. During the nesting phase, nestlings of both species share similar environmental conditions, including those related to parental care. However, magpie nestlings that develop together with cuckoos differ in the social environment from those that grow up in monospecific nests. Thus, consistent differences in the microbiota of host nestlings that do or do not share nests with cuckoos can be interpreted as the results of social interactions with heterospecifics.

**Fig. 1:**
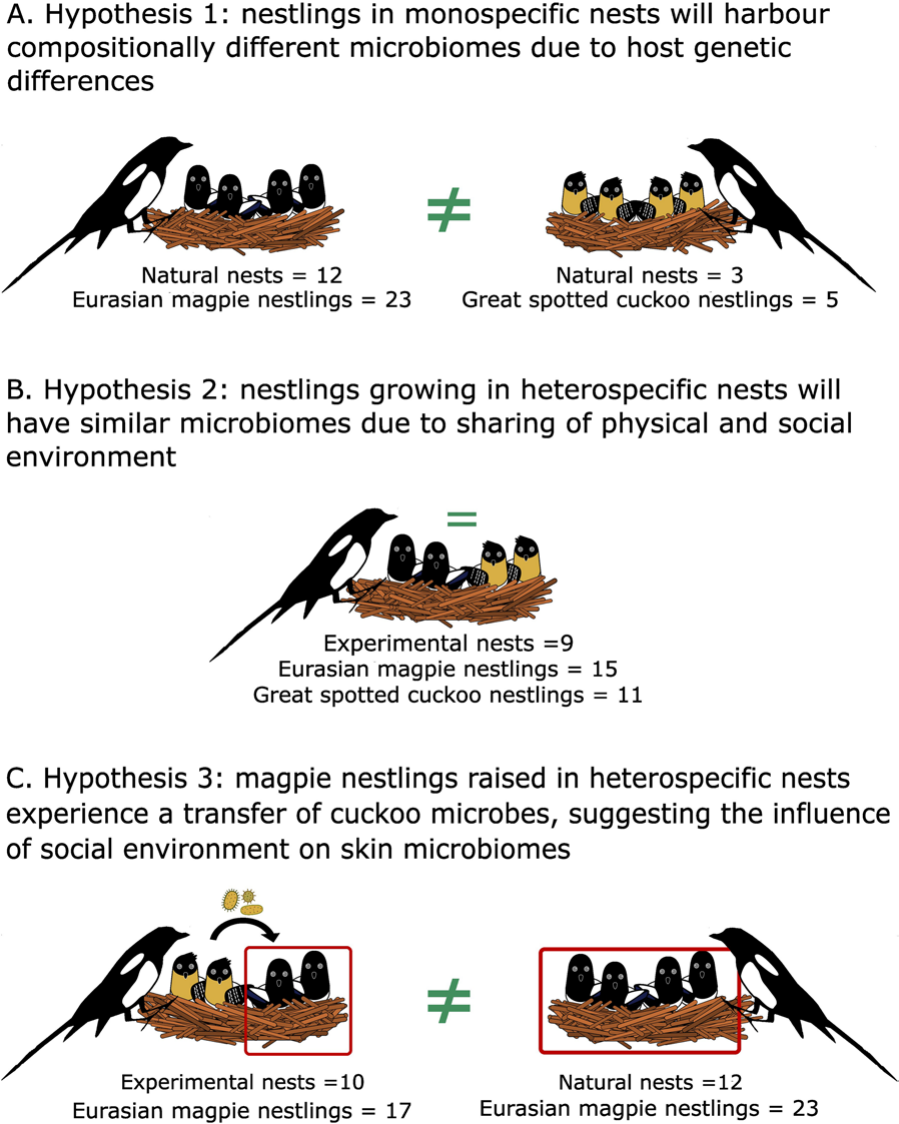
Experimental approach scheme, including the types of magpie nests in the study as well as sample sizes of magpies and cuckoos in each type of nests. The predicted similarities ( =) and differences ( ≠) in microbial composition between nestlings are indicated with green labels

Capitalizing on this natural system, we conducted a cross-fostering experiment, where we manipulated the heterospecific social environments to disentangle the effect of physical and social environments on assembly processes of uropygial gland skin microbiomes (Fig. 1). The cross-fostering approach allowed us to avoid possible biases due to cuckoos choosing nests of particular environmental characteristics [56, 57], and to maximize the number of magpie nests with individuals of both species. Our experimental design allows testing effects of exposing individuals of the same social group (here members of the same species: magpies) to members of a different social group (cuckoos) in the same environment (magpie nests and parents). We characterized the bacterial community of the uropygial gland skin of magpies and cuckoos developing in con- or heterospecific broods via amplicon sequencing of the bacterial 16S rRNA gene. We focused on the uropygial gland skin, as this gland produces a secretion that birds spread onto their feathers and skin while preening [58], and from which several bacterial strains have been isolated [59–67]. The uropygial gland secretion has a species-specific chemical composition [58] which, in contact with skin and other body parts, may act as a substrate that improves the establishment and growth of species-specific bacterial communities [68, 69]. Therefore, we first hypothesised that the skin microbiota of chicks from magpie nests with only magpies or cuckoos (monospecific nests) would vary interspecifically, because of the impact of host intrinsic characteristics on the microbiota (Fig. 1A). Secondly, assuming transmission of microbes between nest-mates in a shared social and physical environment, we expected that chicks growing in heterospecific nests would show reduced interspecific differences (Fig. 1B). Third, given the expected effects of social transmission, we hypothesised that microbial communities would differ between magpie chicks that did or did not grow up with cuckoos (Fig. 1C). We explored these hypotheses in the skin bacterial community as a whole, but also in subsets constituting the core microbiota and a set of potential pathogens.

## Methods

### Study area and fieldwork

Fieldwork was performed during the breeding season of 2018 in the Hoya de Guadix (37°15′N; 3°01′W), a semiarid high-altitude plateau in southern Spain, where a magpie population frequently parasitized by cuckoos is established [70, 71]. The vegetation is sparse, with disperse holm-oak trees (*Quercus rotundifolia*), groves of almond trees (*Prunus dulcis*) and pines (*Pinus halepensis*), where magpies usually build their nests.

Since mid-March, we intensively searched for magpie nests, which allowed us to infer the start of laying (hereafter laying date) and, thus, the expected hatching dates of cuckoo and magpie eggs. Cuckoos might choose to parasitize host nests with characteristics that maximize their reproductive success [56], which might result in biased samples when exclusively using natural parasitized nests, and, thus, highlight the importance of adopting an experimental approach. Moreover, magpie nestlings usually starve in naturally parasitic nests because cuckoo eggs hatch four or more days ahead of those of magpie, which are outcompeted by older cuckoo chicks [72]. To avoid possible bias of naturally parasitized nests, and to maximize the probability that magpies and cuckoo nestlings develop together in the same magpie nests, we cross-fostered cuckoo eggs between magpie nests whenever possible, synchronizing their expected hatching date of cuckoo eggs to the same or one or two days later than that of the magpie eggs. This approach allowed us to maximize the number of heterospecific nests where cuckoo and magpie nestlings developed together [see 19 for a similar experimental approach]. Magpie monospecific nests were simply non-parasitized nests, whereas cuckoo monospecific nests were parasitized magpie nests where natural death of magpie nestlings occurred (Fig. 1). Cuckoo and magpie nestlings develop at different rates [73] and, thus, to sample them at similar developmental stages it was necessary to visit the nests twice. However, we only manipulate the species needed for processing at each visit (either magpie or cuckoo nestlings), leaving the nestlings of the other species in the nest. We collected microbiome samples from 56 nestlings when cuckoo and magpies were, approximately, 15 and 17 days old respectively. Briefly, we sampled skin microbiota of nestlings by rubbing the surface skin of the uropygial gland, including the opening, with a sterile cotton swab (APTACA, ref. 2160, Canelli, Italy) wetted in sterile Phosphate Buffer Saline (PBS, 0.2 M). The swab with the bacterial sample was kept in a sterile microfuge vial with 1 mL of sterile PBS and stored at -18 °C until DNA extraction. At the time of sampling, we also measured tarsus length with a digital calliper (accuracy 0.01 mm), wing length with a ruler (accuracy 1 mm), body mass with a digital scale (accuracy 0.01 g), and gland dimensions (length, width and height) with a digital calliper (accuracy 0.01 mm) as described previously [74].

### DNA extraction and amplicon sequencing

DNA from the sampled bacterial communities was extracted using the FavorPrep™ Blood Genomic DNA Extraction Mini Kit (Favorgen Biotech Crop., Taipei, Taiwan), following this protocol: first, the sample was sonicated for 2 min at 120 Hz to release the bacterial cells from the swab. The swab was then removed, and the PBS with the bacteria was centrifuged at 13,000 rpm for 5 min. The supernatant was discarded, and 180 µl of TES (25 mM Tris–HCl, pH 8, 10 mM EDTA and 10% sucrose), 10 mg/ml of lysozyme and 10 mg/ml of RNase were added to the pellet. Subsequent steps were performed according to the FavorPrep™ protocol. From this extraction, 5 µl were used to perform a PCR reaction to verify the presence of bacterial DNA in the uropygial skin. The PCRs were conducted using the primers B969F (ACGCGHNRAACCTTACC) and BA1406R (ACGGGCRGTGWGTRCAA) [75]. The PCR products were visualized on a 1% agarose gel with electrophoresis. Libraries for paired-end Illumina sequencing were constructed in two steps following Caporaso approach [76] with the bacterial V6-V8 region of the 16S rRNA using the same pair of primers specified above. These primers maximize the amplification of bacteria and reduce non-specific eukaryotic amplifications [77]. Library construction and paired-end sequencing (2 × 300) in the MiSeq (Illumina) platform was carried out at the Institute of Parasitology and Biomedicine "López-Neyra" facilities (IPBLN, Granada, Spain).

### Bioinformatic amplicon data processing

We first processed the amplicon sequences in QIIME2 v2020.6 [78], using default parameters unless stated otherwise. Primer trimming and sequence quality filtering were performed using DADA2 [79], and all sequences were clustered into ASVs (Amplicon Sequence Variants) at 100% similarity and assigned to taxonomy using the Silva 138 database [80]. Due to the primers’ specificity for bacteria, non-bacterial sequences, and sequences identified as mitochondrial or chloroplast, were removed from the ASV table. Contaminant sequences were identified from field (open swabs without sample) and laboratory (extraction and sequencing blanks) negative controls with the “Decontam” package in R [81, 82] using the prevalence method and a threshold of 0.4. Sequences were aligned and a rooted bacterial phylogeny was generated using the method *align-to-tree-mafft-fasttree* in QIIME2. One sample was filtered out due to markedly low reads (2,500). The ASV table was rarefied to the minimum sampling depth (14,877 sequences) using the method *rarefy_even_depth* in the “phyloseq” package [83].

We also identified potential avian pathogens and the core microbiome for each species among the detected ASVs. For the potential avian pathogens, we first ran the FAPROTAX script in python [84], which converts prokaryotic abundance tables (ASV tables) into putative functional abundance profiles. The ASVs considered by FAPROTAX as animal pathogens were searched in the literature to certify avian pathogenicity. Besides, we also used the Pathogen Host Interaction database (PHI-base) [85] and the review published by Benskin et al. in 2009 [86] to search for genera that includes potential known pathogenic bacteria of birds. We use those datasets to build a new ASV table that included potential pathogenic ASVs belonging to genus with available information (Additional file 1). We also calculated the core microbiome using a relative abundance of 0.0001% in at least 50% of the samples in the “phylosmith” package [87] in R v4.0.2 [82]. We did so separately for each species and type of social environment considered (i.e., only magpies, only cuckoos, magpies that grew with cuckoos and cuckoos that developed together with magpies). Then, we created a subset of the ASV table pruning out taxa that did not belong to the core microbiome for each species.

### Statistical analyses

Alpha diversity indexes and beta diversity distance matrixes were calculated in R v4.0.2 [82]. Alpha diversity was calculated with Shannon’s diversity index using “microbiome” package [88], while Faith’s phylogenetic diversity (PD) was computed using “picante” package [89]. Beta diversity matrices were calculated using Bray–Curtis, Jaccard, weighted UniFrac and unweighted UniFrac distances, and PCoA plots were generated with Bray–Curtis distances and visualized using “phyloseq” package [83].

Factors expected to influence alpha and beta diversity indexes were respectively explored in mixed model ANOVAs and PERMANOVAs. The effects of species identity (hereafter, ID) were explored with information from nests where only cuckoo or only magpie nestlings developed (monospecific nests). The models included species ID as fixed factor and the nest ID (nested within, species ID) as the random factor. The effects of species ID were also explored in nests where magpie and cuckoo nestlings develop together (heterospecific nests), but in this case, the statistical model included species ID as the fixed factor and nest ID and the interaction of nest ID with species ID as random factors. The effect of social environment was analysed by comparing magpie nestlings that grew in monospecific nests with those that developed together with cuckoos in heterospecific nests. These models included the social environment (mono- or heterospecific magpie nests) as the fixed factor and nest ID (nested within social environment) as the random factor. Brood size did not significantly explain alpha diversity indexes (Additional file 2) and, thus, was not included as covariable in the statistical models.

We tested which bacterial genera had significant differential abundances among the four types of social environments. We did this by using the *trans_diff* function from “microeco” package [90] in R v4.0.2 [82] with the Wilcoxon Rank Sum method and False Discovery Rate (FDR) adjusted p-values. We conducted differential abundance analyses with the whole ASV table. Finally, we used *betadisper* function in the “vegan” package [91] using spatial median and adjusted biases to analyse the homogeneity of variances among magpies sharing and not sharing nests with cuckoos. The effects of species ID and social environment on alpha and beta diversities of subsets that included potentially pathogenic bacteria, or the core microbiome, were explored in statistical models identical to those described above. ANOVAs were conducted in STATISTICA v.12 [92], while PERMANOVAs were performed with Primer7 v.7.0.17 (PRIMER-e).

## Results

**Fig. 2:**
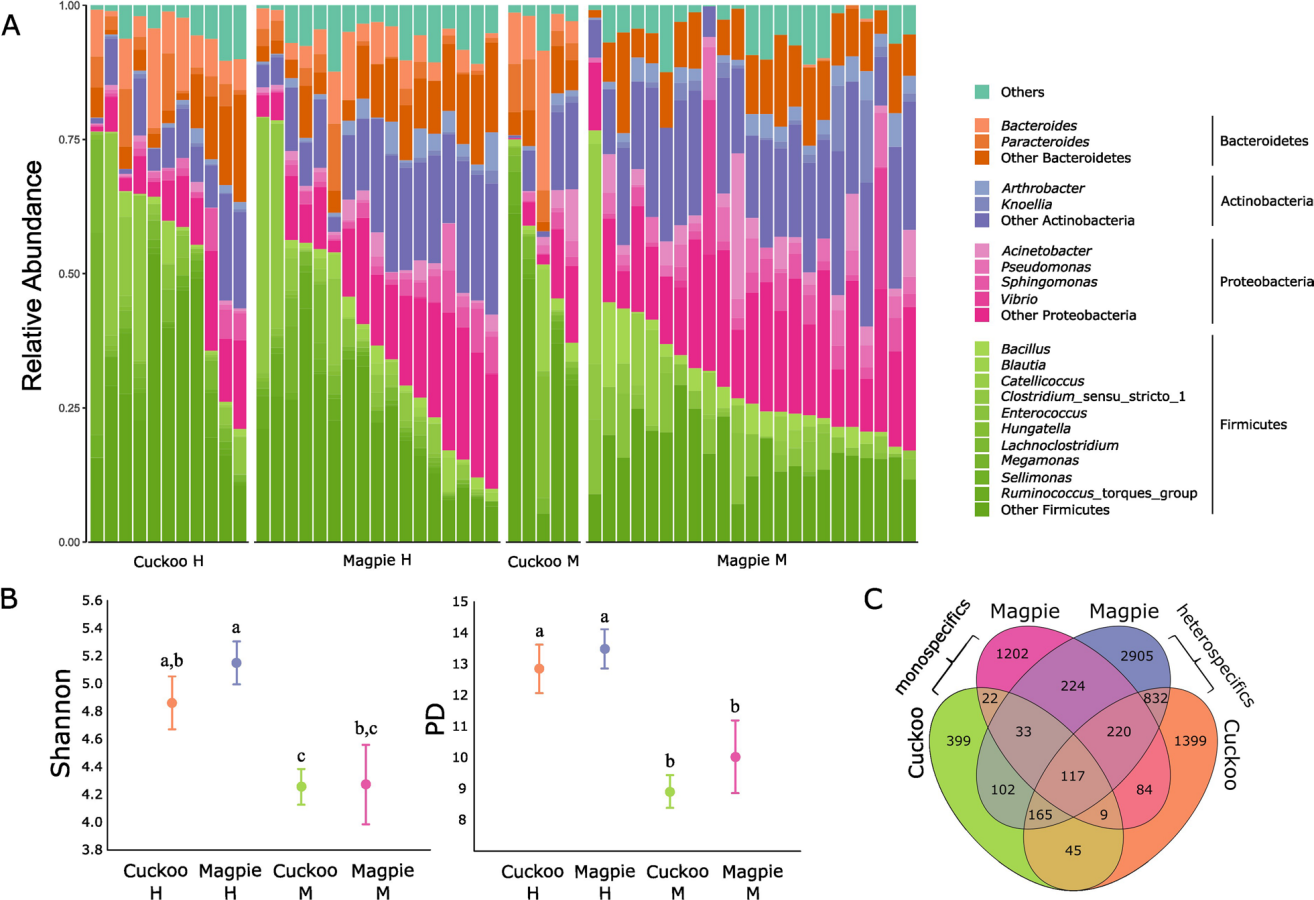
**A** Microbial composition at the phylum and genus levels of uropygial gland skin of great spotted cuckoos and Eurasian magpies from monospecific (M) or heterospecific (H) nests. **B** Least square means (± 95% CI) of alpha diversity indexes (Shannon’s diversity index and Faith’s phylogenetic distance (PD)) estimated for magpies and cuckoos from monospecific (M) or heterospecific (H) nests. **C** Venn diagram showing the number of shared ASVs between different treatment groups

We successfully sequenced 56 nestling samples (40 magpies and 16 cuckoos, for sample sizes see Table 1), from which we obtained 1,950,249 sequences classified into 7,825 ASVs belonging to 21 bacterial phyla. Before rarefaction, each sample had an average of 34,825.88 (SD ± 10,692.12) sequences. Rarefaction led to a reduction in the total number of ASVs to 7,758 (Additional file 3). The whole data set was dominated by Firmicutes (40.1%), Proteobacteria (22.7%), Actinobacteria (18%) and Bacteroidetes (14.2%). Firmicutes dominated in both species, but there were species-specific differences despite the high individual variation. In cuckoos, Bacteroidetes was the second most abundant phylum, followed by Proteobacteria and Actinobacteria, while Proteobacteria was the second most common phylum in magpies, followed by Actinobacteria and Bacteroidetes (Fig. 2A). Moreover, although some bacterial groups appeared in both cuckoo and magpie samples (Fig. 2A), the most abundant genera differed between bird species. *Clostridium* (4.8%), *Enterococcus* (3.8%), *Acinetobacter* (3.3%), and *Pseudomonas* (3.2%) were the most abundant genera in magpie samples, while *Bacteroides* (12%), *Clostridium* (7.2%), *Parabacteroides* (6.4%), and *Lachnoclostridium* (4.6%) were the most abundant bacteria in cuckoo samples (Fig. 2A; Additional file 4).

**Table 1:** Sample sizes (number of nests and nestlings sampled) for each of the four combinations of species (magpies or great spotted (GS) cuckoos and social environment (monospecific or heterospecific)

Group	Sample sizes		Average number of nestlings per nest	Number of nestlings considered after quality filtering
	Nestlings	Nests		
Magpies Monospecific	23	12	1.92	23
Magpies Heterospecific	18	10	1.86	17
GS Cuckoos Monospecific	5	3	1.67	5
GS Cuckoos Heterospecific	11	9	1.25	11

### Microbial diversity

Considering the whole set of ASVs, alpha diversity indexes of magpie and cuckoo samples did not differ significantly (Table 2). That was the case independently of comparing samples from mono- or heterospecific nests (Table 2). However, alpha diversity of magpie samples from heterospecific nests was significantly higher than that of magpie samples from monospecific nests (Table 2, Fig. 2B). Interestingly, magpies growing up in heterospecific nests shared more ASVs with cuckoos than with magpies or cuckoos from monospecific nests (Fig. 2C).

**Table 2:** Results from mixed model ANOVAs exploring the effects of species ID in either, mono- or heterospecific nests, as well as the effect on social environment on the alpha diversity indexes (Shannon’s diversity index and Faith’s phylogenetic diversity (PD)) on bacterial communities of the uropygial gland skin of magpies and great spotted cuckoos. We analysed the effects of species ID and social environment on diversity of the whole bacterial community, the core microbiome and the subset of potentially pathogenic ASVs. Results with associated p-value lower than 0.05 are shown in bold. Fixed *(f)* and random *(rnd)* factors are indicated

		All ASVs				Potentially pathogenic ASVs				Core microbiome			
		MS	F	*df*	*p*	MS	F	*df*	*p*	MS	F	df	*p*
Shannon	*Species effect (monospecific nests)*												
	Species ID *(f)*	< 0.01	0.002	1,13.9	0.962	0.42	2.91	1,14.8	0.109	**2.79**	**11.9**	**1,14.6**	**0.004**
	Nest (Species ID) *(rnd)*	**0.52**	**5.57**	**13,13**	**0.002**	**0.16**	**2.86**	**13,13**	**0.034**	**0.26**	**3.17**	**13,13**	**0.023**
	Error	0.09				0.06				0.08			
	*Species effect (heterospecific nests)*												
	Species ID *(f)*	0.45	1.74	1,8.3	0.223	0.10	0.67	1,8.2	0.435	0.11	0.72	1,8.4	0.419
	Nest *(rnd)*	**1.34**	**4.98**	**8,8**	**0.018**	0.31	2.08	8,8	0.160	**0.87**	**5.42**	**8,8**	**0.014**
	Species ID*Nest *(rnd)*	0.27	3.32	8,8	0.055	**0.15**	**5.96**	**8,8**	**0.010**	0.16	2.51	8,8	0.106
	Error	0.08				0.03				0.06			
	*Effect social environment magpies*												
	Social environment *(f)*	**7.28**	**15.75**	**1,20.5**	** < 0.001**	0.15	0.60	1,20.7	0.445	**1.43**	**5.24**	**1,20.9**	**0.033**
	Nest (Social environment) *(rnd)*	**0.50**	**6.01**	**20,18**	** < 0.001**	**0.27**	**4.97**	**20,18**	** < 0.001**	**0.29**	**3.91**	**20,18**	**0.003**
	Error	0.08				0.05				0.07			
PD	*Species effect (monospecific nests)*												
	Species ID *(f)*	4.66	1.00	1,14.2	0.334	0.03	0.21	1,16.5	0.650	**15.80**	**28.46**	**1,14**	** < 0.001**
	Nest (Species ID) *(rnd)*	**5.33**	**4.28**	**13,13**	**0.006**	0.13	1.45	13,13	0.256	**0.64**	**5.07**	**13,13**	**0.003**
	Error	**1.24**				0.09				0.13			
	*Species effect (heterospecific nests)*												
	Species ID *(f)*	1.59	0.57	1,8.5	0.470	0.04	0.27	1,8.5	0.615	0.02	0.06	1,8.35	0.818
	Nest *(rnd)*	**10.85**	**3.79**	**8,8**	**0.039**	0.17	1.19	8,8	0.407	**1.25**	**4.42**	**8,8**	**0.025**
	Species ID*Nest *(rnd)*	2.86	1.89	8,8	0.193	0.14	1.95	8,8	0.183	0.28	2.57	8,8	0.102
	Error	1.51				0.07				0.11			
	*Effect social environment magpies*												
	Social environment *(f)*	**190.22**	**32.38**	**1,20.8**	** < 0.001**	0.21	1.56	1,21.3	0.226	**24.31**	**37.9**	**1,20.7**	** < 0.001**
	Nest (Social environment) *(rnd)*	**6.57**	**4.60**	**20,18**	** < 0.001**	**0.14**	**2.66**	**20,18**	**0.021**	**0.69**	**5.24**	**20,18**	** < 0.001**
	Error	1.37				0.05				0.13			

**Fig. 3:**
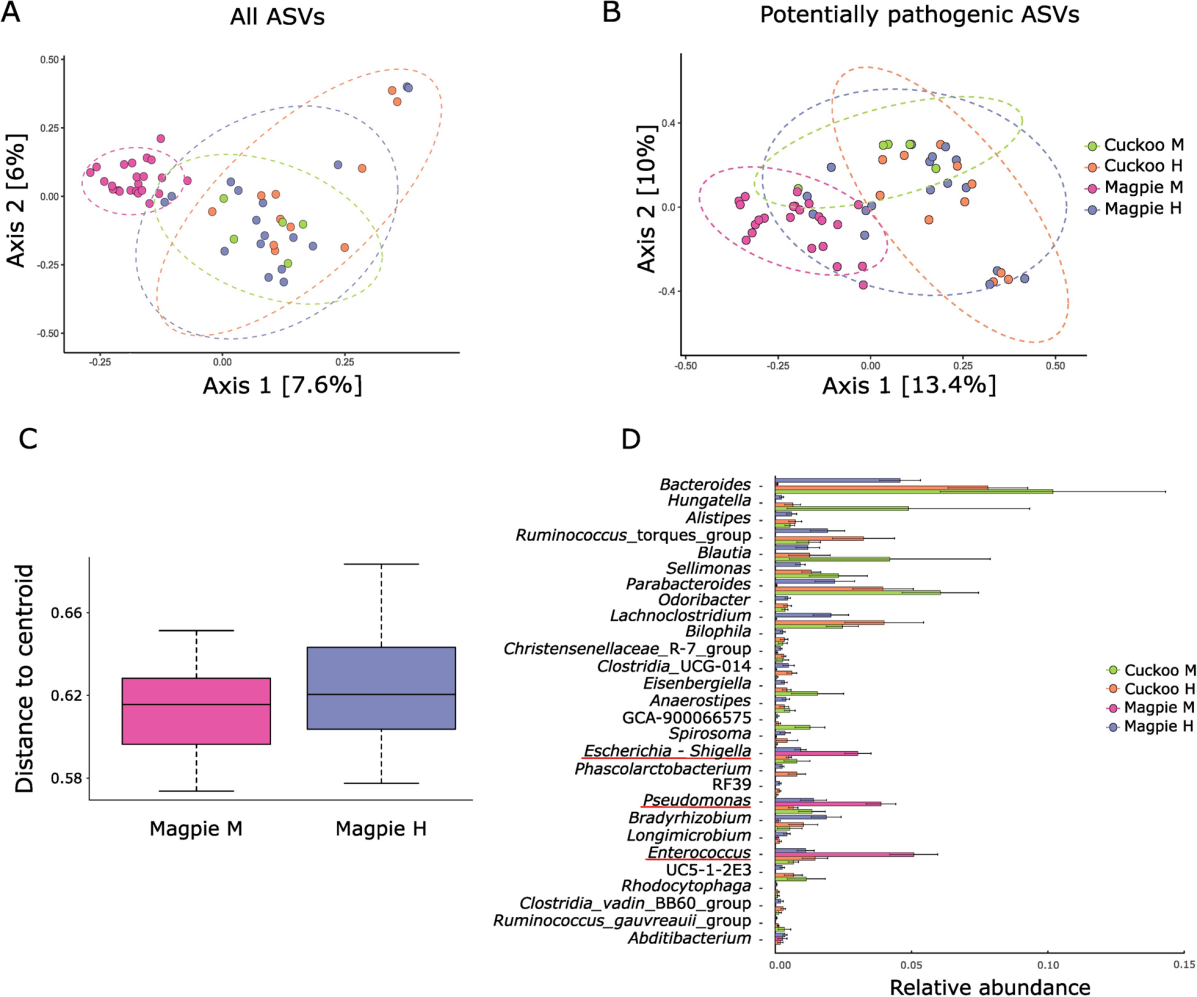
PCoA plots show the microbiome composition based on Bray–Curtis distance matrixes of magpies and great spotted cuckoos from monospecific (M) or heterospecific (H) nests using (**A)** the whole set of ASVs and (**B)** only potentially pathogenic ASVs. Ellipses are 95% confidence interval. (**C)** Box plots showing group dispersions with Bray–Curtis distance matrix (betadisper analyses) of magpies from monospecific (M) or heterospecific (H) nests. (**D)** Differential abundance of genera from the whole dataset of ASVs differing significantly among species (magpies or great spotted cuckoos) and/or social environments (nestlings from monospecific (M) or heterospecific (H) nests). The list indicates  bacterial genera derived from Random Forest analyses with alpha = 0.01. Underlined in red are the potentially pathogenic genera

When considering the beta diversity of cuckoo and magpie samples from monospecific nests, the bacterial communities differed significantly between the two species [except for weighted UniFrac distances (Table 3)], and their 95% confidence interval ellipses in PCoA plots hardly overlap (Fig. 3A). This effect disappeared when comparing samples of magpies and cuckoo nestlings that were raised in the same nest (Table 3), as revealed by overlapping points and 95% confidence intervals in Fig. 3A. Furthermore, regardless of the distance matrix used, the social environment influences the composition of the bacterial community of magpies (Table 3, Fig. 3). However, the individual variation in magpie microbiomes was not associated with social environment (Fig. 3C) when considering Bray–Curtis, Jaccard, or weighted UniFrac distance matrixes (betadisper test; F_1,38_ < 2.64, *p* > 0.109), but it was associated with social environment when considering Unweighted UniFrac (betadisper test; F_1,38_ = 23.21, *p* < 0.001).

**Table 3:** Results from PERMANOVAs analysing beta diversity matrixes of the whole bacterial community, the core microbiome or potentially pathogenic ASVs subsets as dependent variables. The analyses explored the effect of species ID (taking into account mono- and heterospecific nests), and the effect of social environment on the microbial communities of magpie and great spotted cuckoo nestlings. The number of permutations was set to 9999. Results with associated *p*-value lower than 0.05 are shown in bold. Fixed *(f)* and random *(rnd)* factors are indicated

		Βray-Curtis				Jaccard				Unweighted UniFrac				Weighted UniFrac			
		MS	Pseudo-F	*df*	*p*	MS	Pseudo-F	*df*	*p*	MS	Pseudo-F	*df*	*p*	MS	Pseudo-F	*df*	*p*
All ASVs	*Species effect (monospecific nests)*																
	Species ID *(f)*	**0.85**	**1.90**	**1,16.3**	**0.002**	**0.72**	**1.51**	**1,16.9**	**0.009**	**0.42**	**2.35**	**1,16.4**	**0.009**	0.01	2.62	1,15.2	0.091
	Nest (Species ID) *(rnd)*	**0.48**	**1.55**	**13,13**	** < 0.001**	**0.50**	**1.30**	**13,13**	** < 0.001**	**0.19**	**1.47**	**13,13**	** < 0.001**	< 0.01	2.33	13,13	0.099
	Error	0.31				0.38				0.13				< 0.01			
	*Species effect (heterospecific nests)*																
	Species ID *(f)*	0.26	1.06	1,9	0.430	0.34	1.02	1,9	0.485	0.22	1.21	1,9.1	0.282	< 0.01	2.96	1,9.3	0.089
	Nest *(rnd)*	**0.74**	**2.78**	**8,8**	** < 0.001**	**0.69**	**1.99**	**8,8**	** < 0.001**	**0.37**	**1.74**	**8,8**	**0.001**	< 0.01	1.45	8,8	0.271
	Species ID*Nest *(rnd)*	0.24	0.91	8,8	0.730	0.33	0.96	8,8	0.652	0.18	0.85	8,8	0.850	< 0.01	0.69	8,8	0.729
	Error	0.27				0.35				0.21				< 0.01			
	*Effect social environment magpies*																
	Social environment *(f)*	**1.03**	**2.19**	**1,22.2**	** < 0.001**	**0.82**	**1.66**	**1,22.7**	** < 0.001**	**0.93**	**4.20**	**1,22.6**	** < 0.001**	**0.01**	**5.47**	**1,21.8**	**0.006**
	Nest (Social environment) *(rnd)*	**0.49**	**1.57**	**20,18**	** < 0.001**	**0.50**	**1.31**	**20,18**	** < 0.001**	**0.23**	**1.35**	**20,18**	** < 0.001**	< 0.01	1.99	20,18	0.116
	Error	0.31				0.38				0.17				< 0.01			
Potentially pathogenic ASVs	*Species effect (monospecific nests)*																
	Species ID *(f)*	**0.89**	**2.76**	**1,16.6**	**0.009**	**0.78**	**2.00**	**1,17.1**	**0.009**	0.12	0.72	1,16.4	0.404	0.04	0.86	1,16.7	0.365
	Nest (Species ID) *(rnd)*	**0.34**	**1.40**	**13,13**	**0.004**	**0.40**	**1.22**	**13,13**	**0.008**	0.18	1.50	13,13	0.184	0.04	1.37	13,13	0.276
	Error	0.24				0.33				0.01				0.03			
	*Species effect (heterospecific nests)*																
	Species ID *(f)*	0.21	0.93	1,8.8	0.526	0.29	0.96	1,8.9	0.538	0.01	0.43	1,8.6	0.589	0.06	2.83	1,9	0.119
	Nest *(rnd)*	**0.62**	**2.94**	**8,8**	** < 0.001**	**0.64**	**2.13**	**8,8**	** < 0.001**	0.02	1.88	8,8	0.169	**0.10**	**4.31**	**8,8**	**0.006**
	Species ID*Nest *(rnd)*	0.23	1.08	8,8	0.371	0.30	1.01	8,8	0.487	0.02	1.49	8,8	0.298	0.02	0.88	8,8	0.584
	Error	0.21				0.30				0.01				0.02			
	*Effect social environment magpies*																
	Social environment *(f)*	**1.30**	**3.41**	**1,22.1**	** < 0.001**	**1.07**	**2.46**	**1,22.5**	** < 0.001**	0.38	2.15	1,21.6	0.150	0.21	3.11	1,21.1	0.075
	Nest (Social environment) *(rnd)*	**0.39**	**1.66**	**20,18**	** < 0.001**	**0.45**	**1.38**	**20,18**	** < 0.001**	**0.02**	**2.15**	**20,18**	**0.026**	**0.07**	**3.20**	**20,18**	**0.004**
	Error	0.24				0.32				0.01				0.02			
Core microbiome	*Species effect (monospecific nests)*																
	Species ID *(f)*	**1.22**	**3.83**	**1,15.4**	**0.002**	**1.03**	**2.60**	**1,16.1**	**0.002**	**0.40**	**6.80**	**1,15.3**	**0.002**	**0.57**	**13.00**	**1,14.4**	**0.003**
	Nest (Species ID) *(rnd)*	**0.35**	**2.14**	**13,13**	** < 0.001**	**0.42**	**1.65**	**13,13**	** < 0.001**	**0.06**	**2.20**	**13,13**	** < 0.001**	**0.50**	**3.59**	**13,13**	** < 0.001**
	Error	0.16				0.26				0.03				0.01			
	*Species effect (heterospecific nests)*																
	Species ID *(f)*	0.19	1.20	1,8.8	0.311	0.26	1.07	1,8.8	0.419	0.02	0.95	1,9	0.478	0.08	2.64	1,8.5	0.103
	Nest *(rnd)*	**0.66**	**4.55**	**8,8**	** < 0.001**	**0.69**	**3.08**	**8,8**	** < 0.001**	**0.08**	**3.12**	**8,8**	** < 0.001**	**0.12**	**7.87**	**8,8**	** < 0.001**
	Species ID*Nest *(rnd)*	0.16	1.10	8,8	0.356	0.25	1.09	8,8	0.303	0.02	0.89	8,8	0.650	0.03	1.93	8,8	0.079
	Error	0.14				0.23				0.03				0.02			
	*Effect social environment magpies*																
	Social environment *(f)*	**1.47**	**4.24**	**1,21.6**	** < 0.001**	**1.25**	**2.97**	**1,22.0**	** < 0.001**	**0.70**	**12.62**	**1,21.8**	** < 0.001**	**0.22**	**3.50**	**1,20.9**	**0.027**
	Nest (Social environment) *(rnd)*	**0.36**	**2.22**	**20,18**	** < 0.001**	**0.44**	**1.71**	**20,18**	** < 0.001**	**0.06**	**1.99**	**20,18**	** < 0.001**	**0.07**	**4.15**	**20,18**	** < 0.001**
	Error	0.16				0.26				0.03				0.02

Thirty bacterial genera were significantly differentially abundant between groups (Additional file 5). Thirteen, 17 and 21 genera were respectively more abundant in cuckoos from monospecific nests, cuckoos from heterospecific nests, and magpies from heterospecific nests than in magpies from monospecific nests. Magpies from monospecific nests had higher relative abundances of *Enterococcus* than magpies from heterospecific nests, and higher relative abundances of *Escherichia – Shigella* and *Pseudomonas* than magpies and cuckoos from heterospecific nests (Fig. 3D, Additional file 5). Interestingly, magpies in heterospecific nests did not present any differentially abundant genera compared with cuckoos, either from mono- or heterospecific nests.

### Core microbiome

Core microbes with relative abundance higher than 0.0001% and that appeared in 50% of the samples for a species comprised 232 ASVs from 75 genera in six phyla (Additional file 6). For these cores, we detected interspecific differences in alpha and beta diversity between nestlings of the two species from monospecific nests (Tables 2 and 3). Furthermore, and in agreement with the results for all ASVs, bacterial diversity and community composition of birds in heterospecific nests did not differ between species (Tables 2 and 3). In addition, differences in social environment for magpie nestlings were also apparent when exploring the core microbiome (Tables 2 and 3).

### Diversity and composition of potentially pathogenic ASVs

Alpha diversity of potential pathogenic taxa did not differ significantly among magpies and cuckoos when considering either mono- or heterospecific nests (Table 2). Similarly, sharing the nest with heterospecifics did not affect the alpha diversity indexes of magpie samples (Table 2). When looking at beta diversity of communities of potential pathogens, we found statistically significant interspecific differences when comparing monospecific nests and considering Bray–Curtis or Jaccard distance matrices (Table 3). However, this effect disappeared when comparing cuckoo and magpie nestlings that grew up within the same nests (Table 3). Moreover, beta diversity of potential pathogenic bacteria of magpies differed according to social environment when considering Bray–Curtis and Jaccard distance matrixes (Table 3). Furthermore, *Pseudomonas, Escherichia*—*Shigella* and *Enterococcus* were significantly more abundant in magpies that grew up in monospecific nests (Fig. 3D).

## Discussion

By capitalising on the natural associations between magpie hosts and cuckoo brood parasites, we document the influence of social environment on microbial assemblages of the uropygial gland skin of developing magpie and parasitic cuckoo chicks. As expected, we detected species-specific diversity and composition in both complete microbiomes and microbial cores, underlining the role of species-specific factors in shaping microbial assemblages. However, and as predicted, these interspecific differences disappeared when species cohabitated the same nests, implying either social transmission of symbionts among nestlings or transmission of microbes via feeding adults and/or the shared physical environment [c.f., 93]. In heterospecific nests, we observed a change in the magpie microbiota to resemble that of their heterospecific nestmates that most likely imply horizontal transmission of microbes between cuckoos and magpies via their altered social environment. Lastly, we observed a negative impact of social environment on certain candidate pathogenic bacteria in magpies, suggesting potential positive effects of the presence of cuckoos on magpie skin microbiomes.

We found interspecific differences in the uropygial gland skin microbiomes in monospecific nests when using any of the distance matrixes, with the exception of Weighted UniFrac. UniFrac distances are controlled for phylogenetic association of the considered ASVs, and the lack of differences may suggest that the detected interspecific differences for other distance matrices are driven by the relative abundance of phylogenetically closely related ASVs. These interspecific differences cannot only be accounted by vertical transmission of microbes from the biological or foster parents. This is particularly true for cuckoos, as vertical transmission of microbes is restricted to the pre-egg laying period [94, 95]. These differences may thus result from intrinsic factors, such as for example species-specific chemical composition of the uropygial gland secretion [58, 68, 69]. Even during the nestling stage, birds preen their feathers and skin with uropygial secretion [96], and thus the antimicrobial properties of these secretions [97, 98] may prevent specific bacteria from establishing [99–101], and stimulate the growth of other microbial taxa [69]. Consequently, it is likely that the particularities of the uropygial secretion of magpies and cuckoos promote a species-specific selective environment that favours certain microbes to grow on the skin, a possibility worth exploring in the future by testing for promoting or inhibitory effects of the uropygial secretion on the bacterial strains. Another non-exclusive possibility explaining interspecific differences is the potential transfer of species-specific fecal microbes  [19] to the skin of the uropygial gland. Cuckoos have defensive faeces with a strong smell to deter predators [102], which might host particular bacterial taxa that could be responsible for the detected interspecific differences. Some of these chemical-producing bacteria are likely anaerobic bacteria, which would explain the higher prevalence of the anaerobic *Bacteroides*, *Clostridium*, *Parabacteroides*, and *Lachnoclostridium* in cuckoo samples.

In gut microbial communities of cuckoos and magpies, species specificities in cloacal microbiomes are retained in heterospecific nests [19, 21]. In contrast, we found that skin microbiomes converged in the host-parasite species pair. This indicates that the magnitude of the effect of social and shared physical environment varies depending on whether the host-associated microbiomes are external or internal [34, 103]. Despite similarities in the microbial composition (e.g., saliva microbiomes) and diet between cuckoo and magpie nestlings in the same nests [19], species-specific cloacal microbiomes imply internally-maintained digestive tract microbiomes that are resistant to perturbations from the shared environment [21], while skin microbiomes are more susceptible to horizontal transfer.

The evidence for social transmission of microbes stems mainly from the potential transfer of cuckoo-specific microbes to magpies. This unidirectional transfer of microbes from cuckoos to magpies might indicate that the resilience of skin microbiomes to the social environment also varies interspecifically. Given the brood-parasite lifestyle, cuckoos may depend more on *in ovo* vertical transmission of microbes than magpie nestlings [c.f., 104], while having more resistant skin microbiomes to ensure transgenerational transfers of symbionts [c.f., 93]. This skewed opportunity for vertical transmission of microbes in cuckoos may alter host-symbiont associations in this species with potential implications for losses or replacements of microbial symbionts across generations. However, the effects of sharing a nest with heterospecifics might only be temporal, as shown by a cross-fostering experiment on captive zebra finch (*Taeniopygia guttata*) and Bengalese finch (*Lonchura striata domestica*) nestlings [45]. In these cases, effects of foster families on cloacal microbiomes early in the nestling period disappeared in later stages [45]. In the case of the uropygial gland, its secretion might not be fully developed during the nestling stage [58] and, thus, the associated microbiome is likely shaped during the second part of the nesting phase [105], explaining why we detected the expected effect of social environment at the late developmental stage of magpie nestlings. However, to fully grasp the breadth of how early life social environment influences long-term associations and generational transfers of skin symbionts, we need to explore the fate of skin microbes over time during an individual’s life.

The social transfer of potentially pathogenic bacteria contrasted the patterns for non-pathogenic bacteria, where the relative abundances of potentially pathogenic genera *Pseudomonas*, *Escherichia*—*Shigella* and  *Enterococcus* were significantly higher in magpies in monospecific than heterospecific nests. The lower prevalence of these genera in heterospecific nests could be mediated by the parallel increase in the diversity of the skin microbiota of nestlings. This might be because increased microbial diversity provides increased resistance to pathogen colonization [106, 107] and also stimulates the host immune system [11]. Alternatively, the properties of the uropygial secretion, or symbiotic defensive bacteria within the gland of cuckoos, may counter potential pathogens. Even if this might have a positive effect of reduced pathogens on host magpies, it would be unlikely to counter the negative fitness effects of brood parasitism [108]. Nevertheless, because we did not test for pathogenicity of strains, these inferences are only tentative and, to fully understand the implication of social environment on pathogenic taxa, future research is needed to explore the specific bacterial strains with detrimental effects on birds, and how they are distributed among con- and heterospecific nests. Furthermore, to disentangle the mechanisms underlining the observed negative effect of social environment on potentially pathogenic bacteria, we need to isolate these bacterial taxa and conduct co-culture assays with cuckoo and magpie uropygial gland secretions.

## Conclusions

Using a natural host-brood parasite system, we were able to separate the effects of genetic relatedness and shared environment on the skin microbiota from those of interacting individuals, elucidating a role of social environment determining the skin microbiomes of wild birds. Our study implies that skin microbiomes are amenable to horizontal transfer of microbes from the social and the nest environment, but that the magnitude and identities of bacterial genera transferred depend on host ecology. Early-life exposure to heterospecific microbes can thus alter wild bird skin microbiomes, which should influence both short and long-term stability of beneficial and antagonistic symbiotic interactions.

### Supplementary Information

**Additional file 1**.

**Additional file 2**.

**Additional file 3**.

**Additional file 4**.

**Additional file 5**.

**Additional file 6**.

## Data Availability

Amplicon sequences have been uploaded to the SRA archive in GenBank (Accessions PRJNA957771).
