# Supplementary material for: Social environment influences microbiota and potentially pathogenic bacterial communities on the skin of developing birds
Source: Anim Microbiome. 2024 Aug 15;6:47. doi: 10.1186/s42523-024-00327-2 (PMC11325624; doi:10.1186/s42523-024-00327-2)
Supplement: Supplementary file 1 — Additional file 1. [file 42523_2024_327_MOESM1_ESM.docx]

**Additional file 1**. Taxonomy of strains considered potentially pathogenic. We considered as potentially pathogenic strains every bacteria inside a genus described as pathogenic in one of the three sources. Information was extracted from PHI-base [1], FAPROTAX [2] and a review published by Benskin CMH, Wilson K, Jones K and Hartley IR [3].

| **Domain** | **Phylum** | **Order** | **Class** | **Family** | **Genus** | **Species** | **Source** |
| --- | --- | --- | --- | --- | --- | --- | --- |
| Bacteria | Firmicutes | Clostridia | Clostridiales | Clostridiaceae | Clostridium_sensu_stricto_1 | | PHI database |
| Bacteria | Firmicutes | Bacilli | Lactobacillales | Enterococcaceae | Enterococcus |  | Benskin et al. 2009 |
| Bacteria | Firmicutes | Clostridia | Clostridiales | Clostridiaceae | Clostridium_sensu_stricto_1 | uncultured_bacterium | PHI database |
| Bacteria | Firmicutes | Clostridia | Clostridiales | Clostridiaceae | Clostridium_sensu_stricto_1 | uncultured_organism | PHI database |
| Bacteria | Firmicutes | Clostridia | Clostridiales | Clostridiaceae | Clostridium_sensu_stricto_1 | Clostridium_celatum | PHI database |
| Bacteria | Firmicutes | Clostridia | Clostridiales | Clostridiaceae | Clostridium_sensu_stricto_7 | uncultured_Clostridium | PHI database |
| Bacteria | Firmicutes | Clostridia | Clostridiales | Clostridiaceae | Clostridium_sensu_stricto_9 | | PHI database |
| Bacteria | Proteobacteria | Gammaproteobacteria | Enterobacterales | Enterobacteriaceae | Escherichia-Shigella |  | PHI database |
| Bacteria | Proteobacteria | Gammaproteobacteria | Pasteurellales | Pasteurellaceae |  |  | Benskin et al. 2009 |
| Bacteria | Proteobacteria | Gammaproteobacteria | Pasteurellales | Pasteurellaceae | |  | Benskin et al. 2009 |
| Bacteria | Proteobacteria | Gammaproteobacteria | Pseudomonadales | Pseudomonadaceae | Pseudomonas | Pseudomonas_caeni | Benskin et al. 2009 |
| Bacteria | Proteobacteria | Gammaproteobacteria | Pseudomonadales | Pseudomonadaceae | Pseudomonas |  | Benskin et al. 2009 |
| Bacteria | Firmicutes | Bacilli | Staphylococcales | Staphylococcaceae | Staphylococcus |  | Benskin et al. 2009 |
| Bacteria | Firmicutes | Clostridia | Clostridiales | Clostridiaceae | Clostridium_sensu_stricto_1 | Clostridium_perfringens | PHI database |
| Bacteria | Proteobacteria | Gammaproteobacteria | Coxiellales | Coxiellaceae | Coxiella |  | FAPROTAX |
| Bacteria | Verrucomicrobiota | Chlamydiae | Chlamydiales | cvE6 | cvE6 | uncultured_Chlamydia | FAPROTAX |
| Bacteria | Proteobacteria | Gammaproteobacteria | Pasteurellales | Pasteurellaceae | Haemophilus |  | FAPROTAX |
| Bacteria | Firmicutes | Bacilli | Lactobacillales | Listeriaceae | Listeria |  | Benskin et al. 2009 |
| Bacteria | Proteobacteria | Gammaproteobacteria | Enterobacterales | Yersiniaceae | Serratia | Serratia_myotis | Benskin et al. 2009 |
| Bacteria | Proteobacteria | Gammaproteobacteria | Enterobacterales | Yersiniaceae | Serratia |  | Benskin et al. 2009 |
| Bacteria | Firmicutes | Bacilli | Staphylococcales | Staphylococcaceae | Staphylococcus | Staphylococcus_simulans | Benskin et al. 2009 |
| Bacteria | Firmicutes | Bacilli | Lactobacillales | Streptococcaceae | Streptococcus |  | Benskin et al. 2009 |
| Bacteria | Firmicutes | Bacilli | Lactobacillales | Streptococcaceae | Streptococcus | Streptococcus_alactolyticus | Benskin et al. 2009 |
| Bacteria | Firmicutes | Bacilli | Lactobacillales | Streptococcaceae | Streptococcus | Streptococcus_equi | Benskin et al. 2009 |
| Bacteria | Proteobacteria | Gammaproteobacteria | Enterobacterales | Yersiniaceae | Yersinia |  | Benskin et al. 2009 |
| Bacteria | Chlamydiota | Chlamydiae | Chlamydiales | Parachlamydiaceae | |  | FAPROTAX |

**References**

1. Urban M, Cuzick A, Seager J, Wood V, Rutherford K, Venkatesh SY, De Silva N, Martinez MC, Pedro H, Yates AD: **PHI-base: the pathogen–host interactions database.** *Nucleic acids research* 2020, **48:**D613-D620.

2. Louca S, Parfrey LW, Doebeli M: **Decoupling function and taxonomy in the global ocean microbiome.** *Science* 2016, **353:**1272-1277.

3. Benskin CMH, Wilson K, Jones K, Hartley IR: **Bacterial pathogens in wild birds: a review of the frequency and effects of infection.** *Biological Reviews* 2009, **84:**349-373.
