# Supplementary material for: Social environment influences microbiota and potentially pathogenic bacterial communities on the skin of developing birds
Source: Anim Microbiome. 2024 Aug 15;6:47. doi: 10.1186/s42523-024-00327-2 (PMC11325624; doi:10.1186/s42523-024-00327-2)
Supplement: Supplementary file 2 — Additional file 2. [file 42523_2024_327_MOESM2_ESM.docx]

**Additional file 2**. Results from mixed model ANOVAs exploring the effects of species ID in either, mono- or hetero-specific nests, as well as the effect on social environment on the alpha diversity indexes (Shannon index and Faith’s phylogenetic diversity (PD)) on magpies bacterial communities. The analyses are controlled for the number of nestlings in the nest. We analysed the effects of species ID and social environment on the whole bacterial community and on the subsets of core microbiome and potentially pathogenic ASVs isolated from the uropygial gland skin of magpies and great spotted cuckoos. Results with associated p-value lower than 0.05 are shown in bold.

|  |  |  | **All ASVs** | | | **Potentially pathogenic ASVs** | | | **Core microbiome** | | |
| --- | --- | --- | --- | --- | --- | --- | --- | --- | --- | --- | --- |
|  |  |  | F | *df* | p | F | *df* | p | F | df | p |
| **Shannon** | *Species effect (monospecific nests)* | |  |  |  |  |  |  |  |  |  |
|  |  | Brood size | 0.17 | 1,12.4 | 0.687 | 0.09 | 1,12.7 | 0.770 | 0.49 | 1,13.4 | 0.495 |
|  |  | Species ID | 0.27 | 1,12.1 | 0.612 | 2.11 | 1,12.1 | 0.172 | 0.02 | 1,12.2 | 0.880 |
|  |  | Nest (Species ID) | **6.03** | **12,13** | **0.001** | **3.06** | **12,13** | **0.028** | 1.51 | 12,13 | 0.235 |
|  | *Species effect (hetero-specific nests)* | |  |  |  |  |  |  |  |  |  |
|  |  | Brood size | 1.87 | 1,6.8 | 0.215 | 0.15 | 1,6.8 | 0.710 | 0.07 | 1,6 | 0.798 |
|  |  | Species ID | 0.86 | 1,8.9 | 0.379 | 0.80 | 1,8.5 | 0.397 | 0.99 | 1,7.2 | 0.350 |
|  |  | Nest | **4.01** | **7,7.6** | **0.038** | 2.27 | 7,7.8 | 0.139 | 0.94 | 7,7.2 | 0.531 |
|  |  | Species ID*Nest | 3.32 | 8,8 | 0.055 | **5.96** | **8,8** | **0.010** | 1.71 | 8,8 | 0.233 |
|  | *Effect social environment magpies* | |  |  |  |  |  |  |  |  |  |
|  |  | Brood size | **5.61** | **1,19.15** | **0.029** | 0.68 | 1,19.2 | 0.420 | 3.78 | 1,19.8 | 0.066 |
|  |  | Social environment | **13.40** | **1,18.8** | **0.002** | 0.44 | 1,18.8 | 0.515 | 2.97 | 1,18 | 0.102 |
|  |  | Nest (Social environment) | **5.45** | **19,18** | **<0.001** | **5.10** | **19,18** | **<0.001** | 1.10 | 19,18 | 0.421 |
| **PD** | *Species effect (monospecific nests)* | |  |  |  |  |  |  |  |  |  |
|  |  | Brood size | 0.42 | 1,12.5 | 0.529 | 0.10 | 1,13.3 | 0.758 | 2.06 | 1,12.4 | 0.173 |
|  |  | Species ID | 1.43 | 1.12.1 | 0.255 | 0.01 | 1,12.2 | 0.922 | **11.38** | **1,12.1** | **0.005** |
|  |  | Nest (Species ID) | **4.64** | **12,13** | **0.005** | 1.57 | 12,13 | 0.217 | **4.78** | **12,13** | **0.004** |
|  | *Species effect (hetero-specific nests)* | |  |  |  |  |  |  |  |  |  |
|  |  | Brood size | 0.37 | 1,6.8 | 0.562 | 3.62 | 1,6 | 0.106 | 0.19 | 1,6.6 | 0.676 |
|  |  | Species ID | 0.42 | 1,8.6 | 0.534 | 0.04 | 1,7.3 | 0.854 | 4.56 | 1,6.9 | 0.071 |
|  |  | Nest | **4.59** | **7,7.3** | **0.029** | 0.85 | 7,7.3 | 0.582 | 4.88 | 7,6.2 | 0.033 |
|  |  | Species ID*Nest | 1.89 | 8,8 | 0.193 | 1.95 | 8,8 | 0.183 | 0.73 | 8,8 | 0.665 |
|  | *Effect social environment magpies* | |  |  |  |  |  |  |  |  |  |
|  |  | Brood size | 2.83 | 1,19.2 | 0.109 | 0.19 | 1,19.3 | 0.670 | 1.68 | 1,19.4 | 0.210 |
|  |  | Social environment | **28.51** | **1,18.8** | **<0.001** | 2.05 | 1,18.6 | 0.168 | **41.34** | **1,18.5** | **<0.001** |
|  |  | Nest (Social environment) | **4.67** | **19,18** | **<0.001** | **2.74** | **19,18** | **0.019** | 2.07 | 19,18 | 0.064 |
